# Prognosis and risk factors for reocclusion after mechanical thrombectomy

**DOI:** 10.1002/acn3.50999

**Published:** 2020-03-10

**Authors:** Weili Li, Jiayue Ding, Xueqin Sui, Zhifeng Qi, Longfei Wu, Chenghe Sun, Kangxiang Ji, Qingfeng Ma, Xunming Ji, Ke Jian Liu

**Affiliations:** ^1^ Cerebrovascular Diseases Research Institute Xuanwu Hospital Capital Medical University Beijing China; ^2^ Department of Neurology Xuanwu Hospital Capital Medical University Beijing China; ^3^ Advanced Center of Stroke Beijing Institute for Brain Disorders Beijing China; ^4^ Department of General Medicine Affiliated Hospital of Weifang Medical University Weifang Shandong Province China; ^5^ Department of Neurosurgery Xuanwu Hospital Capital Medical University Beijing China; ^6^ Department of Pharmaceutical Sciences College of Pharmacy University of New Mexico Health Sciences Center Albuquerque New Mexico

## Abstract

**Objective:**

This study evaluates reocclusion prognostic outcomes and explores reocclusion risk factors after mechanical thrombectomy (MT) in Chinese stroke patients.

**Methods:**

Altogether, 614 patients with AIS with successful recanalization after MT were recruited in this study and divided into the reocclusion and the non‐reocclusion group depending on the 24‐h imaging results after MT. Differences between the two groups were compared including 24‐h and 7‐day National Institutes of Health Stroke Scale (NIHSS) scores, 90‐day modified Rankin scale(mRS) scores, good prognosis (mRS:0–2) rates, incidence of intracranial hemorrhage, and 90‐day mortality.

**Results:**

Forty‐four (7.2%) patients experienced reocclusion within 24 h. Compared with the non‐reocclusion group, patients in the reocclusion group had higher 24‐h (15 vs. 13) and 7‐day (15 vs. 9) NIHSS scores, 90‐day mRS scores (4 vs. 3), and 90‐day mortality rates (34.1% vs. 18.6%); lower rates of good prognosis (13.6% vs. 9.3%); and a higher incidence of early neurological deterioration (36.4% vs. 14.7%). Age, internal carotid artery occlusion (ICA), intravenous thrombolysis (IVT), number of thrombectomy passes, stent implantation, and levels of D‐dimer (adjusted odds ratio and 95% confidence interval: 0.97, 0.94–0.99; 2.40, 1.10–5.23; 2.21, 1.05–4.66; 2.60, 1.04–6.47; 0.25, 0.09–0.67; and 1.06, 1.01–1.12, respectively) were independently associated with 24‐h reocclusion.

**Interpretation:**

The prognosis of reocclusion after MT was poor. Timely evaluation of these factors including age, D‐dimer, ICA occlusion, IVT, number of passes, and stent implantation and appropriate intervention could reduce the incidence of reocclusion for Chinese stroke patients.

## Introduction

After five high‐evidence clinical trials were published in 2015, mechanical thrombectomy (MT) became the recommended strategy for treating acute ischemic stroke (AIS).^1^, ^2^, ^3^, ^4^, ^5^ Although recanalization plays a pivotal role in improving the outcomes of patients treated with MT in some instances, it is also associated with complications, such as symptomatic intracranial hemorrhage (sICH) and reocclusion. Many researchers are currently studying sICH mechanisms and focusing on ways to identify early symptoms and intervene at early stages.^6^, ^7^ Although research on early reocclusion is rare, the incidence of early symptomatic reocclusion is not lower than that of sICH in stroke patients treated with intravenous thrombolysis (IVT)^8^; it is reported to be 14–24% along with the incidence of reocclusion after IVT.^9^, ^10^ Studies have reported that MT has resulted in recanalization rates over 80%, but the rate of reocclusion within 24 h after MT is approximately 3–9%.^11^, ^12^ Reocclusion after MT can lead to disease deterioration and increased mortality and disability. Therefore, preventing reocclusion after revascularization is necessary in clinical settings. Although the risk factors associated with reocclusion occurrence have not been completely established, a previous study found that stroke severity and severe carotid stenosis were independently associated with early occlusion after IVT.^10^ Additionally, Marto et al. reported that reocclusion after MT was independently related to statin preconditioning, the occlusion site, atherosclerosis, and residual thrombus or stenosis^13^; however, this was a single‐center study that only included European populations. It is already known that environmental factors and racial differences are closely related to stroke incidence^14^; hence, we speculate that there may be reocclusion differences between races in different regions. Environmental and genetic factors may also influence postoperative reocclusion. Therefore, to identify the factors contributing to postoperative reocclusion in Chinese stroke patients, we analyzed patients who had undergone MT at our center in the past. This study aimed to investigate the risk factors and clinical outcomes for reocclusion in MT patients to guide clinical strategies, reduce the incidence, and improve the prognosis of reocclusion.

## Methods

### Patient selection

**Figure 1:**
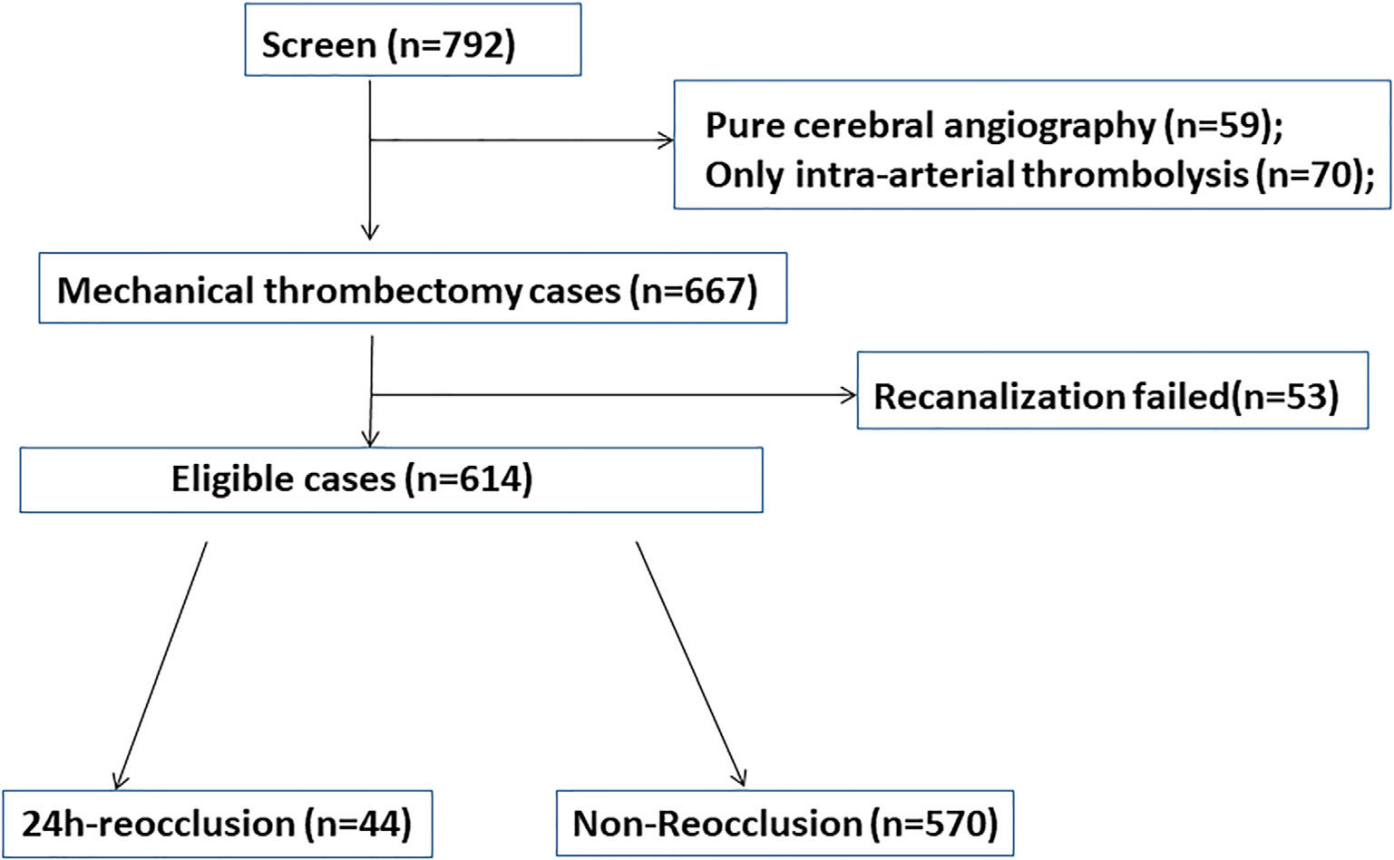
Flow chart of patient grouping. A total of 667 patients were screened for MT from 792 cases, and 614 patients were finally eligible. Forty‐four patients were assigned to the reocclusion group and 570 patients were assigned to the non‐reocclusion group.

Patients with AIS were retrospectively investigated underwent emergency intravascular treatment at Xuanwu Hospital, Capital Medical University from January 2014 to July 2019. Inclusion criteria are as follows: age ≥18 years; a National Institutes of Health Stroke Scale (NIHSS) score ≥6; Alberta Stroke Program Early CT (ASPECT) score ≥6; lesion vessel in the internal carotid artery (ICA), middle cerebral artery (MCA) M1 segment, or basilar artery; the time of stroke onset was within 6 h or more than 6 h but computed tomography (CT) perfusion imaging showed obvious penumbra; intracranial hemorrhage was excluded from brain CT findings; patients provided informed consent; patients underwent MT with successful recanalization (modified Thrombolysis in Cerebral Ischemia [mTICI]: 2b/3). Exclusion criteria were as follows: serious organic diseases, such as heart, liver, and kidney diseases; intracranial or other tumors; international normalized ratio (INR) > 1.7 or platelet count < 100 × 10^9^; and a survival period < 90 days. The selection process is presented in Figure 1.

### Study design

This single‐center retrospective study was approved by the ethics committee of Xuanwu Hospital, Capital Medical University. Patients were divided into reocclusion and non‐reocclusion groups based on the incidence of reocclusion within the 24 h after MT. Transcranial doppler (TCD) and carotid ultrasound were routinely used for postoperative examinations of patients to confirm reocclusion; CT angiography (CTA) or magnetic resonance angiography (MRA) were used with individual patients to confirm vascular reocclusion only when TCD or carotid ultrasound could not provide data for a clear diagnosis.

TCD was performed with the TC8080 TCD ultrasonic diagnostic instrument (Nicolet, Germany) using a 1.6 Hz pulse wave Doppler probe for detection. The MCA and internal carotid siphon segment were observed through the sacral window, and the basilar artery was observed through the occipital window. CTA or MRA were performed to confirm the vascular occlusion if the sacral window was poor.

Magnetic resonance imaging (MRI) was performed at 3 tesla using a standard 32‐channel head coil (MAGNETOM Verio, Siemens Healthcare, Erlangen, Germany). Time‐of‐flight MRA parameters were: repetition time/echo time = 22/4 msec; field of view = 170 × 170 mm^2^. CTA was performed using a 64‐row detector CT scanner, Discovery HD 750 (GE Healthcare, Milwaukee, Wisconsin) with an administration of iodinated contrast (Iopromide 370; Bayer, Germany). Clinical data, including demographic information, previous history of stroke, imaging presentation, clinical outcomes, and experimental indicators were recorded.

### Clinical outcome assessment

The safety and efficacy of MT was also investigated in this study. MT efficacy was evaluated using the 24‐h and 7‐day NIHSS scores, 90‐day modified Rankin Scale (mRS) scores, and 90‐day good outcome rates. A good outcome was defined with an mRS score of 0–2. Safety outcomes comprised early neurological deterioration (END), ICH, sICH, pneumonia, and deep vein thrombosis (DVT) rates within 1 week after MT, and 90‐day mortality. Patients were diagnosed with END if the NIHSS score increased by ≥4 points within 24–48 h,^15^ and sICH was determined according to the European Cooperative Acute Stroke Study (ECASS)‐III diagnostic criteria (ICH was confirmed by a CT or MRI scan within 22–36 h with a ≥4 point NIHSS score increase; ICH is the main cause of neurological deterioration).^1^ Postoperative pneumonia was confirmed by clinical symptoms and lung CT. DVT was diagnosed using a lower extremity venous ultrasound. According to the mTICI criteria, scores ≥ 2b were defined as successful revascularization and scores < 2b were defined as recanalization failure.^16^ Additionally, the etiology of ischemic stroke was classified according to the Trial of Org 10172 in Acute Stroke Treatment (TOAST) classification that includes large‐artery atherosclerosis, cardioembolism, and other types.

### Statistical analysis

Data were analyzed using SPSS 22.0 (IBM Corporation, Armonk, NY) Continuous variables are presented as the mean ± standard deviation or median (interquartile range). Comparisons between the reocclusion and the non‐reocclusion groups were performed using Student’s *t*‐tests for normally distributed data and Mann–Whitney *U* tests for non‐normally distributed data. Categorical variables are expressed as *n* (%) and were analyzed using Pearson *χ* ^2^ or Fisher’s exact tests. *P* < 0.05 was considered statistically significant. To further remove the effect of confounders, multivariate regression models were used. Results were displayed as the adjusted odds radio (aOR) and 95% confidence interval (95% CI). Variables with *P* < 0.2 in the univariate analysis were included in the regression model to determine the risk factors for postoperative reocclusion.

## Results

### Baseline characteristics

A total of 667 patients were treated with MT from January 2014 to July 2019. Thrombectomy was not successful in 53 patients; therefore, 614 were included in this study. Among them, 559 were diagnosed via TCD, 40 were diagnosed using MRA; and 15 were diagnosed by CTA. Forty‐four of the 614 (7.2%) patients experienced reocclusion within 24 h after MT. These patients were assigned to the reocclusion group; the remaining 570 patients (92.8%) who did not suffer reocclusion were assigned to the non‐reocclusion group. The mean patient age in the reocclusion and non‐reocclusion groups was 57.3 ± 10.3 and 63.6 ± 12.4 years (*P* = 0.001), respectively. Males accounted for 72.7% of the participants in the reocclusion group and 75.8% of the participants in the non‐reocclusion group (*P* = 0.649). The median NIHSS scores of the two groups were 15 (12–20) in the reocclusion group and 16 (12–23) in the non‐reocclusion group (*P*> 0.05); the median ASPECT score of both groups was 9 (8–10). There were no significant differences in the onset‐to‐recanalization time (median: 499 vs. 440 min, *P* = 0.471); puncture‐to‐recanalization time (median: 65 vs. 68 min, *P* = 0.463); general anesthesia rate (45.5% vs. 51.4%, *P* = 0.447); or postoperative tirofiban utilization rate (56.8% vs. 62.8%, *P* = 0.429) (Table 1).

**Table 1:** Characteristics of the patients at baseline.

Variable	Reocclusion (*n* = 44)	Non‐Reocclusion (*n* = 570)	*P* value
Age – year	57.3 ± 10.3	63.6 ± 12.4	0.001
Male sex – no. (%)	32 (72.7)	432 (75.8)	0.649
Atrial fibrillation – no. (%)	6 (13.6)	140 (24.6)	0.101
Diabetes mellitus – no. (%)	13 (29.5)	146 (25.6)	0.566
Hypertension – no. (%)	33 (75)	403 (70.7)	0.545
Previous ischemic stroke – no. (%)	14 (31.8)	167 (29.3)	0.724
Statin pretreatment	4 (9.1)	136 (23.9)	0.024
Antiplatelet pretreatment	5 (11.4)	153 (26.8)	0.024
NIHSS^a^ score (IQR)	15 (12–20)	16 (12–23)	0.411
ASPECTS^b^ on baseline CT (IQR)	9 (8–10)	9 (8–10)	0.273
Treatment with IVT – no. (%)	21 (47.7)	173 (30.4)	0.017
Occlusion site – no. (%)			
ICA	19 (43.2)	156 (27.4)	0.025
MCA	18 (40.9)	230 (40.4)	0.942
BA	7 (15.9)	184 (32.3)	0.024
Type of TOAST – no. (%)			
Cardiogenic embolism	6 (13.6)	125 (21.9)	0.196
large‐artery atherosclerosis	33 (75)	415 ( 72.8)	0.752
Others	5 (11.4)	30 (5.1)	0.093
On awakening	10 (22.7)	92 (16.1)	0.258
Process measures – min			
Median time from stroke onset to reperfusion (IQR)	499 (342–603)	440 (331–620)	0.471
Median time from femoral puncture to reperfusion (IQR)	65 (39–101)	68 (40–118)	0.463
Number of devices passes			
>2	13 (29.5)	50 (8.8)	<0.001
≦2	31 (77.3)	520 (90.4)	
Stent implantation^c^ – no. (%)	9 (20.5)	205 (36.0)	0.037
General anesthesia	20 (45.5)	293 (51.4)	0.447
Treated with eptifibatide	25 ( 56.8)	358 (62.8)	0.429
Laboratory test within 24 h of admission			
GLU (mmol/L)	7 (6.5–10.1)	7.3 (6.0–9.6)	0.297
LDL‐C (mmol/L)	2 (2.0–3.5)	2.5 (2.0–3.1)	0.787
FIB (g/L)	3 (2.6–3.7)	3.1 (2.6–3.7)	0.873
D‐Dimer (mmol/L)	2 (0.8–4.7)	0.8 (0.4–1.9)	<0.001
Blood platelets (mmol/L)	2 (167–241)	20 (171–236)	0.991

a Scores on the National Institutes of Health Stroke Scale (NIHSS) range from 0 to 42, with higher scores indicating more severe neurologic deficits.

b The alberta stroke program early computed tomography score (ASPECTS) is a measure of the extent of stroke. Scores range from 0 to 10, with higher scores indicating fewer early ischemic changes.

c Stent implantation included only intracranial stents, including the Solitaire, Apollo, and Neroform stents. Carotid stent implantation was excluded.

### Effectiveness outcomes

**Figure 2:**
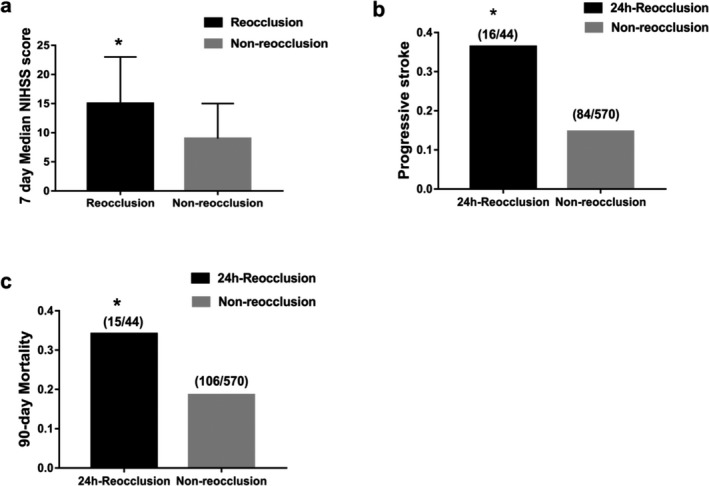
Differences in 7‐day NIHSS scores, early progressive stroke, and 90‐day mortality between the two group. (A) The NIHSS scores of patients in the reocclusion group were significantly higher than those of patients in the non‐reocclusion group at 7 days (15 vs. 9, *P* = 0.001). (B) The incidence of early progressive stroke in the reocclusion group was significantly higher than that in the non‐occlusion group (36.4% vs. 14.7%, *P* < 0.001). (C) The 90‐day mortality rate in the 24‐h reocclusion group was also significantly higher than that in the non‐reocclusion group (34.1% vs. 18.6%, *P* = 0.013).

**Figure 3:**
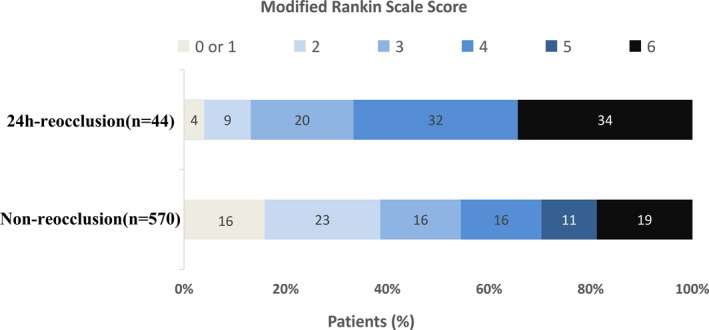
Modified Rankin scale score distribution at 90 days. The distribution of modified Rankin scale (mRS) scores. mRS Scores range from 0 to 6, with 0 indicating no symptoms, 1 no clinically significant disability, 2 slight disability (patient is able to look after own affairs without assistance but is unable to carry out all previous activities), 3 moderate disability (patient requires some help but is able to walk unassisted), 4 moderately severe disability (patient is unable to attend to bodily needs without assistance and unable to walk unassisted), 5 severe disability (patient requires constant nursing care and attention), and 6 death. The proportion of patients with good prognosis (mRS:0‐2) was significantly lower in the reocclusion group (13.6%) than that in the non‐reocclusion group (39.3%, *P* = 0.032). No patients in the reocclusion group had an mRS score of 5.

The overall MT effectiveness was analyzed in 614 patients. The 24‐h NIHSS score was 15 [6–26], the 7‐day NIHSS score was 9 [3–15], the 90‐day mRS score was 3 [2–5], and the good prognosis rate at 90 days was 37.5% (230/614). The 24‐h NIHSS score was significantly higher in the reocclusion group than that in the non‐occlusion group (15 [12–30] vs. 13 [6–23], *P* = 0.015). The 7‐day NIHSS scores in the reocclusion group (15 [9–23]) were significantly higher than those in the non‐occlusion group (9 [13–15], *P* = 0.001) (Table 2, Fig. 2). The 90‐day mRS scores were significantly higher in the reocclusion group (4 [3–6]) than those in the non‐reocclusion group (3 [2–5], *P* = 0.036). The 90‐day good outcome rate was significantly lower in the reocclusion group (13.6%) than that in the non‐reocclusion group (39.3%, *P* = 0.001) (Table 2, Fig. 3). The logistic regression analysis (adjusted for age, sex, stent implantation, intravenous thrombolysis, number of thrombolysis passes, NIHSS score on admission, onset to recanalization time, and TOAST type) revealed that 24‐h reocclusion was negatively correlated with good 90‐day prognoses (aOR: 0.187, 95% CI: 0.04–0.87; *P* = 0.032) (Table 2).

**Table 2:** Comparison of complications and prognosis after MT.

Outcomes	Reocclusion (*n* = 44)	Non‐reocclusion (*n* = 570)	AdjustedOR^a^ (95%CI)	*P* value
24‐h NIHSS score	15 (12–30)	13 (6–23)	Beta	0.015
7‐day NIHSS score	15 (9–23)	9 (3–15)	Beta	0.001
mRS score at 90 days	4 (3–6)	3 (2–5)	Beta	0.036
mRS 0–2 – no. (%)	6 (13.6)	224 (39.3)	0.187 (0.04–0.87)	0.032
Early neurological deterioration	16 (36.4)	84 (14.7)	8.22 (2.48–27.29)	0.001
ICH at 24 h	14 (31.8)	155 (27.2)	0.54 (0.12–2.52)	0.432
sICH at 24 h	3 (6.8)	45 (7.9)	0.31 (0–3.5)	0.345
Pneumonia	28 (63.6)	313 (54.9)	2.17 (0.72–6.57)	0.172
DVT	14 (31.8)	90 (15.8)	2.32 (0.83–6.44)	0.107
Death at 90 days	15 (34.1)	106 (18.6)	8.48 (2.4–30)	0.001

a Adjusted for age, gender, stent implantation, intravenous thrombolysis, number of thrombolysis, NIHSS score on admission, onset to recanalization time.

### Safety outcomes

The safety results for all MT patients showed that the total END incidence was 16.3% (100/614), the ICH incidence was 27.5% (169/614), the sICH incidence was 7.8% (48/614), and the 90‐day mortality rate was 19.7% (121/614). The END incidence in the reocclusion group (16/44 [36.3%]) was significantly higher than that in the non‐reocclusion group (84/570 [14.7%], *P* < 0.001) (Table 2, Fig. 2). After adjusting for age, gender, stent implantation, number of intravenous thrombolysis passes, NIHSS scores on admission, onset to recanalization time, and TOAST type, the logistic regression analysis revealed that reocclusion was an independent risk factor for END (aOR: 8.22, 95% CI: 2.48–27.29; *P* = 0.001). Also, the 90‐day mortality rate in the reocclusion group was significantly higher than that in the non‐reocclusion group (34.1% vs. 18.6%, *P* = 0.013) (Table 2, Fig. 2). Meanwhile, postoperative reocclusion was independently associated with the 90‐day mortality rate (aOR: 8.48, 95% CI: 2.40–30; *P* = 0.001) (Table 2).

There were no significant differences in ICH (31.8% vs. 27.2%; aOR: 0.54, 95% CI: 0.12–2.52; *P* = 0.432), sICH (6.8% vs. 7.9%; aOR: 0.31, 95% CI: 0–3.5; *P* = 0.345), pneumonia (63.6% vs. 54.9%; aOR: 2.17, 95% CI: 0.72–6.57; *P* = 0.172), or DVT (31.8% vs. 15.8%; aOR: 2.32, 95% CI: 0.83–6.44; *P* = 0.107) incidence between the two groups (Table 2).

### Risk factor analysis

The univariate regression analysis was initially performed to identify risk factors with *P* < 0.2. Univariate analysis results revealed that age (unadjusted OR [uOR]: 0.96, 95% CI: 0.94–0.99; *P* = 0.001), statin pretreatment (uOR: 0.29, 95% CI: 0.10–0.83; *P* = 0.021), antiplatelet pretreatment (uOR: 0.32, 95% CI: 0.12–0.84; *P* = 0.020), IVT (uOR: 2.1, 95% CI: 1.1–3.89; *P* = 0.022), ICA occlusion (uOR: 2.54, 95% CI: 1.34–4.81; *P* = 0.004), number of thrombectomy passes (uOR: 4.32, 95% CI: 2.04–8.76; *P* < 0.001), stent implantation (uOR: 0.47, 95% CI: 0.22–1.0; *P* = 0.049), D‐dimer levels (uOR: 1.05, 95% CI: 0.99–1.11: *P* = 0.11), and large‐artery atherosclerosis (uOR: 1.83, 95% CI: 0.98–3.41; *P* = 0.058) were associated with reocclusion (Table 3).

**Table 3:** Regression analysis of risk factors for reocclusion after MT.

Variable	Unadjusted OR (95%CI)	Adjusted OR (95%CI)	Unadjusted/adjusted *P*
Age, year	0.96 (0.94–0.99)	0.97 (0.94–0.99)	0.001/0.020
Statin pretreatment	0.29 (0.10–0.83)	0.41 (0.08–2.02)	0.021/0.27
Antiplatelet pretreatment	0.32 (0.12–0.84)	0.47 (0.10–2.17)	0.020/0.33
Treatment with IVT	2.1 (1.1–3.89)	2.21 (1.05–4.66)	0.022/0.036
ICA occlusion	2.54 (1.34–4.81)	2.40 (1.10–5.23)	0.004/0.029
Number of devices passes	4.23 (2.04–8.76)	2.60 (1.04–6.47)	<0.001/0.04
Stent implantation	0.47 (0.22–1.0)	0.25 (0.09–0.67)	0.049/0.006
D‐Dimer (mmol/L)	1.05 (0.99–1.11)	1.06 (1.01–1.12)	0.11/0.029
Large‐artery atherosclerosis	1.83 (0.98–3.41)	1.54 (0.85–2.77)	0.058/0.152

Next, risk factors with significance levels of *P* < 0.2 in the univariate regression analysis were included in a logistic regression analysis. Results from this analysis revealed that Age, ICA occlusion, IVT, number of thrombectomy passes, stent implantation, and levels of D‐dimer [adjusted odds ratio(aOR) and 95% confidence interval: 0.97, 0.94–0.99; 2.40, 1.10–5.23; 2.21, 1.05–4.66; 2.60, 1.04–6.47; 0.25, 0.09–0.67; and 1.06, 1.01–1.12, respectively] were independently associated with 24‐h reocclusion. Furthermore, there may be a negative association between age and 24‐h reocclusion (aOR < 1). However, statin and antiplatelet pretreatment and large‐artery atherosclerosis were not significantly associated with 24‐h reocclusion (*P* > 0.05) in the multivariate regression model (Table 3).

## Discussion

Although reocclusion is infrequent after MT, once occlusion recurs, it will gravely affect the patient's functional prognosis.^12^, ^13^, ^17^, ^18^, ^19^, ^20^ Due to the low incidence of reocclusion, there are relatively few current related studies. Qureshi et al. evaluated 56 endovascular treatment patients in 2008; early reocclusion was only found in five cases (9%).^17^ Another study of 40 cases of carotid tandem occlusions with simultaneous stenting and MT found nine cases with reocclusion (28.1%).^18^ The study of Marto et al. included 473 successful recanalization patients, of which 28 (6.6%) had reocclusion after MT.^13^ Mosimann found that reocclusion occurred in 16 of 711 cases (2.3%),^12^ and our study found the same to occur in 44 of 614 (7.2%). The risk factors for reocclusion are not identical in each study. Hidaka's study suggested that reocclusion was associated with endothelial injury after MT.^20^ Mosimann thought it was related to high platelet levels upon admission, residual thrombus fragments, and atherosclerotic stenosis.^12^ Marto believed that statin pretreatment, the occlusion site, more complex procedures, the atherosclerotic cause, and residual thrombus or stenosis were associated with reocclusion.^13^

The innovation of the present research lies in the following aspects. First, a large sample size was utilized that could reduce bias. Second, the Chinese population was studied; at present, there are no reports on reocclusion in the Chinese population after a thrombectomy. Third, this study confirms the results of previous studies and presents new risk factors such as age, IVT, and D‐dimer. Hence, these findings are helpful for future studies and postoperative reocclusion predictions.

This study showed that early reocclusion corresponded with increased NIHSS scores at 24 h and 7 days as well as the 90‐day mRS scores, reduced the incidence of good prognoses at 90 days, and increased the risk of END and mortality. This is consistent with previous studies.

Similar to previous studies,^14^ this study also shows that ICA occlusions and the number of passes were associated with reocclusion. Meanwhile, the present study reveals that stent implantation was a protective factor for reocclusion. This conclusion may be an important basis for emergency stenting in patients with atherosclerotic stenosis. Moreover, atherosclerotic stenosis is an independent risk factor for reocclusion that has been demonstrated in multiple studies.^12^, ^13^, ^19^ Although the present univariate regression analysis also found a correlation between atherosclerosis and reocclusion, the multivariate analysis did not indicate statistical significance, perhaps due to the influence of confounding factors. Inconsistent with Marto's research, statin therapy was not designated as a protective factor for early reocclusion.

Interestingly, this study yielded three new findings. First, IVT was an independent risk factor for reocclusion after MT. Previous studies have found that early reocclusion is one of the most common complications after IVT, and the incidence of reocclusion following IVT is 14–24%.^9^, ^10^ Research by Heo et al. suggests that early reocclusion often occurred after IVT.^21^ Platelet mediated‐thrombotic mechanisms maybe play a key role in rethrombosis after thrombolytic‐induced clot lysis.^22^ These findings indicate that IVT can dissolve the thrombus to recanalize the occluded blood vessels, but it is also a risk factor for postoperative reocclusion. Current studies show that IVT can cause fibrin deposition and platelet aggregation that may result in thrombosis (especially shortly after thrombolysis).^9^, ^10^ These results suggest IVT combined with MT maybe not be better than direct MT. In 2018, the Changhai Hospital of China initiated an ongoing direct MT study (Clinical Trials.gov Identifier: NCT03469206). The purpose of this study is to verify whether direct MT is more effective than MT and IVT combined treatment; we look forward to its results. The second novel finding from this study was that D‐dimer elevation was an independent risk factor for reocclusion. A D‐dimer is a specific degradation product of fibrin; elevated plasma D‐dimer levels indicate enhanced fibrinolytic activity. Enhanced fibrinolytic activity signifies that the body's system is in a hypercoagulable state that is likely to lead to thrombosis. Previous studies have confirmed that elevated plasma D‐dimer levels can predict END,^23^ and the common cause of END is reocclusion.^10^ Therefore, D‐dimer levels are speculated to be associated with reocclusion; the present results confirm this assumption. These findings suggest that the postoperative detection of D‐dimer levels can predict the risk of reocclusion after MT.

The present study also found a negative correlation between age and reocclusion. In contrast, Mosimann^12^ reported that there was no significant difference in age between the reocclusion and non‐reocclusion. groups. The present findings may attribute to a self‐resistant mechanism of the body. The younger the patient, the more obvious the defense response of the body; therefore, they are more prone to inflammation and platelet aggregation. Moreover, the number of reocclusion cases is small and may lead to bias. Therefore, the results need to be further verified.

There were multiple limitations of this study. First, this was a retrospective study; the effects of confounding factors could not be balanced though a multivariate regression was conducted. Next, most of the present postoperative reocclusion results were confirmed mainly through TCD. Only 9% of patients were identified by MRA or CTA. However, an ultrasound itself has certain limitations. Although studies have reported good diagnostic consistency between TCD, CTA, and MRA, both specificity and sensitivity are around 90%.^24^, ^25^, ^26^ Furthermore, a prospective large sample cohort study should be conducted to validate the findings of this study.

## Conclusion

The reocclusion prognosis after MT is poor. Reocclusion‐related factors including age, D‐dimer, ICA occlusion, IVT, number of passes and stent implantation, timely identification as well as appropriate intervention have important clinical significance as they may help reduce the risk of reocclusion and improve long‐term prognoses.

## Conflict of Interest

All authors declare that there is no conflict of interest.

## Author Contributions

Weili Li, Xunming Ji, and Ke Jian Liu contributed to the conception and design of the study. Weili Li, Zhifeng Qi, Longfei Wu, Chenghe Sun, Kangxiang Ji, and Qingfeng Ma contributed to the acquisition and analysis of the data. Weili Li, Jiayue Ding, Xueqin Sui, Xunming Ji, and Ke Jian Liu contributed to manuscript drafting and revision.
